# PPA2-associated sudden cardiac death: extending the clinical and allelic spectrum in 20 new families

**DOI:** 10.1038/s41436-021-01296-6

**Published:** 2021-08-16

**Authors:** Anne Guimier, Melanie T. Achleitner, Anne Moreau de Bellaing, Matthew Edwards, Loïc de Pontual, Kirti Mittal, Kyla E. Dunn, Megan E. Grove, Carolyn J. Tysoe, Clémantine Dimartino, Jessie Cameron, Anil Kanthi, Anju Shukla, Florence van den Broek, Diptendu Chatterjee, Charlotte L. Alston, Charlotte V. Knowles, Laura Brett, Jan A. Till, Tessa Homfray, Paul French, Georgia Spentzou, Noha A. Elserafy, Kate S. Lichkus, Bindu P. Sankaran, Hannah L. Kennedy, Peter M. George, Alexa Kidd, Saskia B. Wortmann, Dianna G. Fisk, Tamara T. Koopmann, Muhammad A. Rafiq, Jason D. Merker, Sumith Parikh, Priyanka Ahimaz, Robert G. Weintraub, Alan S. Ma, Christian Turner, Carolyn J. Ellaway, Liza K. Phillips, David R. Thorburn, Wendy K. Chung, Sajel L. Kana, Ona M. Faye-Petersen, Michelle L. Thompson, Alexandre Janin, Karen McLeod, Ruth McGowan, Robert McFarland, Katta M. Girisha, Deborah J. Morris-Rosendahl, Anna C. E. Hurst, Claire L. S. Turner, Robert M. Hamilton, Robert W. Taylor, Fanny Bajolle, Christopher T. Gordon, Jeanne Amiel, Johannes A. Mayr, Kit Doudney

**Affiliations:** 1 INSERM U1163, Université de Paris, Institut Imagine, Paris, France; 2 Service de Génétique, Hôpital Necker Enfants Malades, APHP, Paris, France; 3 Department of Pediatrics, Paracelsus Medical University Salzburg, Salzburg, Austria; 4 Unité médico-chirurgicale de cardiologie pédiatrique, Hôpital Necker Enfants Malades, APHP, Paris, France; 5 Clinical Genetics and Genomics Laboratory, Royal Brompton and Harefield NHS Trust, London, UK; 6 Translational Medicine program and the Genetics & Genome Biology Program, Peter Gilgan Centre for Research and Learning, The Hospital for Sick Children, Toronto, Canada; 7 Children’s Heart Center, Stanford Children’s Health, Palo Alto, CA USA; 8 Stanford Medicine Clinical Genomics Program, Stanford, CA USA; 9 Exeter Genomics Laboratory, Royal Devon and Exeter NHS Foundation Trust, Exeter, UK; 10 Department of Medical Genetics, Kasturba Medical College, Manipal Academy of Higher Education, Manipal, India; 11 Wellcome Centre for Mitochondrial Research, Translational and Clinical Research Institute, Faculty of Medical Sciences, Newcastle University, Newcastle upon Tyne, UK; 12 Paediatric Cardiology, Royal Brompton and Harefield NHS Trust, London, UK; 13 West of Scotland Centre for Genomic Medicine, Queen Elizabeth University Hospital, Glasgow, UK; 14 The Royal Hospital for Children, Glasgow, UK; 15 Department of Pediatrics, Columbia University, New York, NY USA; 16 Genetic Metabolic Disorders Service, The Children’s Hospital at Westmead, Sydney Children’s Hospital Network, Sydney, NSW Australia; 17 Department of Psychological Medicine, University of Otago, Christchurch, New Zealand; 18 Pathogene, Christchurch, New Zealand; 19 Clinical Genetics New Zealand, Christchurch, New Zealand; 20 Amalia Children’s Hospital, Radboudumc, Nijmegen, The Netherlands; 21 Department of Pathology, School of Medicine, Stanford, CA USA; 22 Departments of Pathology and Laboratory Medicine & Genetics, Lineberger Comprehensive Cancer Center, University of North Carolina School Medicine, Chapel Hill, NC USA; 23 Mitochondrial Medicine Center, Neuroscience Institute, Cleveland Clinic, Cleveland, OH USA; 24 The Royal Children’s Hospital Melbourne, Melbourne, VIC Australia; 25 Department of Clinical Genetics, Western Sydney Genetics Program, The Children’s Hospital at Westmead, Sydney, NSW Australia; 26 Disciplines of Genomic Medicine and Child and Adolescent Health, University of Sydney, Sydney, Australia; 27 Heart Centre for Children, The Children’s Hospital at Westmead, Sydney, Australia; 28 SA Pathology, Department of Genetics and Molecular Pathology, Adelaide, SA Australia; 29 University of Adelaide, Adelaide, SA Australia; 30 Murdoch Children’s Research Institute, Royal Children’s Hospital, Melbourne, VIC, Australia; 31 Department of Paediatrics, University of Melbourne, Melbourne, VIC, Australia; 32 Victorian Clinical Genetics Services, Murdoch Children’s Research Institute, Royal Children’s Hospital, Melbourne, VIC, Australia; 33 Division of Clinical Genetics and Metabolism, Nicklaus Children’s Health System, Miami, FL USA; 34 Florida International University, Miami, FL USA; 35 Department of Pathology, University of Alabama at Birmingham, Birmingham, AL USA; 36 Hudson Alpha Institute for Biotechnology, Huntsville, AL USA; 37 Laboratoire de Cardiogénétique Moléculaire, Service de Biochimie et Biologie Moléculaire, Hospices Civils de Lyon, Lyon, France; 38 Institut NeuroMyoGène, Université Claude Bernard Lyon 1, Lyon, France; 39 Department of Genetics, University of Alabama at Birmingham, Birmingham, AL USA; 40 Department of Clinical Genetics, Royal Devon and Exeter NHS Foundation Trust, Exeter, UK; 41 Centre for Postgraduate Nursing Studies and the Department of Pathology and Biomedical Science, University of Otago Christchurch, Otautahi, New Zealand

## Abstract

**Purpose:**

Biallelic hypomorphic variants in *PPA2*, encoding the mitochondrial inorganic pyrophosphatase 2 protein, have been recently identified in individuals presenting with sudden cardiac death, occasionally triggered by alcohol intake or a viral infection. Here we report 20 new families harboring *PPA2* variants.

**Methods:**

Synthesis of clinical and molecular data concerning 34 individuals harboring five previously reported *PPA2* variants and 12 novel variants, 11 of which were functionally characterized.

**Results:**

Among the 34 individuals, only 6 remain alive. Twenty-three died before the age of 2 years while five died between 14 and 16 years. Within these 28 cases, 15 died of sudden cardiac arrest and 13 of acute heart failure. One case was diagnosed prenatally with cardiomyopathy. Four teenagers drank alcohol before sudden cardiac arrest. Progressive neurological signs were observed in 2/6 surviving individuals. For 11 variants, recombinant PPA2 enzyme activities were significantly decreased and sensitive to temperature, compared to wild-type PPA2 enzyme activity.

**Conclusion:**

We expand the clinical and mutational spectrum associated with PPA2 dysfunction. Heart failure and sudden cardiac arrest occur at various ages with inter- and intrafamilial phenotypic variability, and presentation can include progressive neurological disease. Alcohol intake can trigger cardiac arrest and should be strictly avoided.

## INTRODUCTION

The role of pyrophosphatase 2 (PPA2) in mitochondrial disease has recently emerged with the discovery of biallelic *PPA2* variants that cause a partial loss of gene function (hypomorphs) within families affected by recurrent sudden cardiac death in siblings. [1, 2] These two publications reported eight hypomorphic variants segregating within seven families. The spectrum of clinical presentations included sudden unexpected death in children before the age of 2 years, mitochondrial disease leading to death in infants aged between 1 month and 2 years, sudden cardiac arrest following the ingestion of small amounts of alcohol in teenagers, and adults reporting acute sensitivity to alcohol. When available, cardiac histology or magnetic resonance image (MRI) frequently showed evidence of myocardial fibrosis.

Three further reports detailed two families affected by recurrence of sudden death in childhood with individuals harboring novel combinations of biallelic *PPA2* variants [3, 4] and an isolated case. [5] In the first study [3], which focused on identification of the genetic basis of 66 childhood-onset cardiomyopathies from Finland, the authors described two affected sibs who both died of dilated cardiomyopathy at 8 and 5 months of age. Necropsies reported dilation of the left ventricle, focal fibrosis, and inflammatory infiltrates with acute myocyte loss. The second study [4] reported two affected sibs who died unexpectedly at 12 and 10 months of age. Both were diagnosed as sudden unexpected death in infancy (SUDI), attributed to a possible cardiac arrhythmia. Autopsy performed in one child was noncontributive to determining etiology. A third study [5] reported a male who suffered from stridor due to vocal cord hypomotility and died of SUDI at 5 weeks of age. Necropsy showed no dilated or hypertrophic cardiomyopathy.

PPA2 is a mitochondrial-located pyrophosphatase that hydrolyzes inorganic pyrophosphate (PPi), generated by numerous nucleotide-dependent reactions, into two orthophosphate molecules. How inorganic pyrophosphatase deficiency at the cellular level can lead to heart or global multiorgan dysfunction is not well understood. We hypothesized that accumulation of PPi beyond a certain threshold could impact the regulation of mitochondrial inner membrane potential and lead to chronic ADP build-up with consequences in energy-consuming organs. Ingestion of alcohol could act as a trigger by increasing the stress in heart tissue, leading to arrhythmia and cardiac arrest. [1, 2]

Here we present a series of 34 previously unreported individuals (all but one affected) from 20 families and add 12 novel pathogenic *PPA2* variants to the 10 previously published. Functional assays in *E.coli* were performed for all nine of the novel missense variants and two previously reported. [1, 4] We expand the spectrum of clinical presentations to include fetal cardiomyopathy with cerebral malformations and also progressive neurological symptoms in affected living adults. Finally, we highlight the role of alcohol consumption and viral illness as triggers of sudden cardiac arrest.

## MATERIALS AND METHODS

### Genetic testing

Cases (34) and their families (20) were collected via international collaboration. Exome, genome, or cardiac disease gene panel sequencing was performed according to approved ethical and institutional protocols and with informed consent from all families, in different independent research or diagnostic laboratories worldwide, using standard protocols and validated capture, sequencing and variant calling pipelines.

### In silico analysis of *PPA2* variants

AlamutVisual v2.11 (alamut.interactive-biosoftware.com) was used for visualizing variants on the human *PPA2* transcript and protein (accession numbers NM_176869.3 and NP_789845.1, respectively) and for predicting consequences of a splice site variant. American College of Medical Genetics and Genomics/Association for Molecular Pathology (ACMG/AMP) guidelines [6] were used for classification of the novel variants, in concert with guidelines for the classification of variants in rare disease: https://www.acgs.uk.com/quality/best-practice-guidelines/#VariantGuidelines.[7] Clustal Omega version 1.2.4 multiple sequence alignment analysis was carried out using the following protein sequences: *Homo sapiens* NP_789845.1 inorganic pyrophosphatase 2; *Mus musculus* AAH11417.1 pyrophosphatase (inorganic) 2; *Gallus gallus* XP_004941113.1 inorganic pyrophosphatase 2; *Xenopus tropicalis* CAJ83623.1 inorganic pyrophosphatase 2; *Danio rerio* XP_005159941.1 inorganic pyrophosphatase 2; *Drosophila melanogaster* NP_001246494.1 NUF3; *Saccharomyces cerevisiae* 8PRK_A Chain A, Protein (inorganic pyrophosphatase); *Arabidopsis thaliana* CAC19853.1 inorganic pyrophosphatase.

### Functional analysis of *PPA2* variants

*PPA2* complementary DNA (cDNA) was cloned into the plasmid pRSETB (ThermoFisher). Thirteen missense variants were inserted into the construct by site-directed mutagenesis of wild-type *PPA2* [1] (Supplementary Table S1). Incorporation of the variants was confirmed by Sanger sequencing of clones, which were subsequently transformed into the *E. coli* expression strain BL21(DE3) pLysS. Transformed cells were cultured in LBAC (LB + 100 mg/ml ampicillin and 50 mg/ml chloramphenicol) media at 37 °C overnight. After dilution of the cell suspension to an OD^600^ of ~0.2, they were further incubated at 37 °C for 3 hours. The expression of *PPA2* was initiated by addition of 0.5 mmol/l IPTG for 4 hours. Subsequently, the protein was purified from sonicated cell homogenates with HisPur cobalt spin columns (ThermoFisher). [1] For steady-state PPA2 protein concentration comparisons between wild-type and mutated forms of the PPA2 enzyme, Ponceau S staining and Western blotting were carried out using a human PPA2 antibody (Abcam, ab177935; and Supplementary Figure S1). [1] The amount of recombinant protein to be used for enzyme activity measurements was determined by visualization of bands upon Ponceau S staining and Western blotting. Enzyme activity was quantified by measuring absorption of the colorimetric product of phosphate and ammonium heptamolybdate at 620 nm in 96 well plate format with a plate reader at 37 °C. Phosphate concentration was calculated using a standard curve measuring phosphate concentrations ranging from 0.01 µM to 1 M. Differing pyrophosphate concentrations ranging from 0 mM to 0.2 mM were tested, and additionally, enzyme activity was determined at various temperatures ranging from 25 °C to 50 °C. [1] Error bars show the standard error of the mean (SEM).

### Respiratory chain enzyme activities

The activities of respiratory chain enzymes were measured for one affected individual spectrophotometrically in tissue supernatants of cardiac and skeletal muscle (Supplementary Table S2), as previously described. [8]

## RESULTS

### Clinical outcomes

**Fig. 1:**
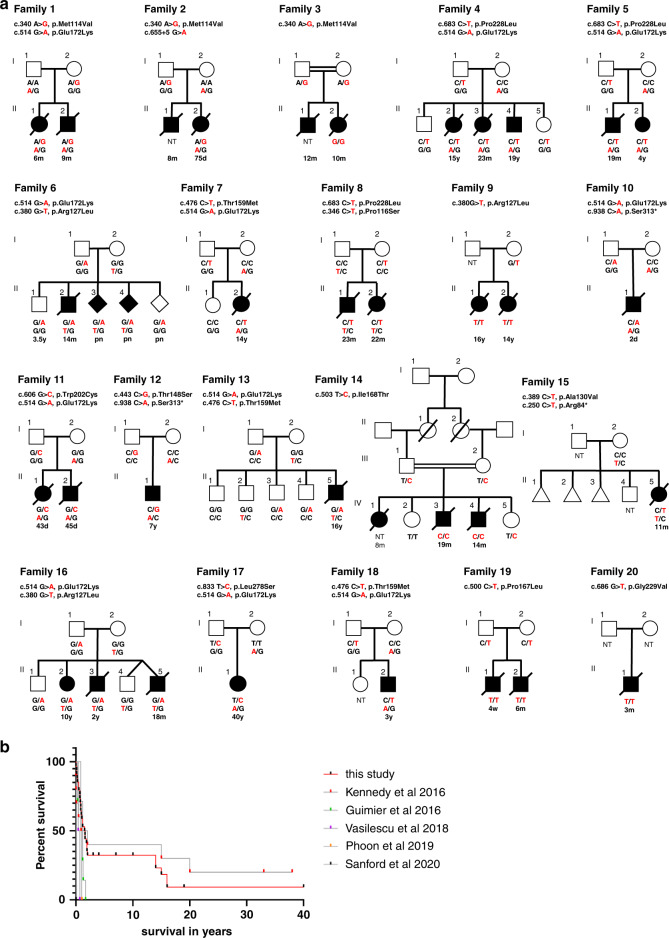
Pedigree structures and survival curves. (**a**) Twenty families with biallelic *PPA2* variants. Variants are marked in red text. Age at death (or current age) is listed under the genotype. d days, m months, pn prenatal diagnosis, NT not tested. Symbols: triangle (miscarriage), diamond (sex unknown). (**b**) Kaplan–Meier survival curves. Kaplan–Meier curves displaying survival data from five previous studies and this cohort. Black and colored marks indicate living individuals (preceded by horizontal lines) or deceased individuals (steps down). Larger steps indicate more individuals who died at a given age. The curve in red summarizes the 34 individuals from this study (six surviving; 28 deceased). Two prenatal individuals of family 6 are not included in this figure because the pregnancies were terminated.

We report 34 individuals from 20 previously unreported families (Fig. 1a) with at least one member affected by rapidly progressive cardiac failure or sudden unexplained death and harboring biallelic *PPA2* variants. Recurrence in siblings was observed in 12 of the 20 families. Key clinical data from the 20 families and from the previously published families are summarized in Table 1. Detailed clinical findings and molecular analyses for the 20 newly identified families are presented in Supplementary Table S3 and as Supplementary Case Reports.

**Table 1:** Summary of major clinical data of the cohort and other cases in the literature.

	This cohort	Kennedy et al. 2016 [1]	Guimier et al. 2016 [2]	Vasilescu et al. 2018 [3]	Phoon et al. 2019 [4]	Sanford et al. 2020 [5]	Total
Number	34^a^	10	7	2	2	1	56
Number of families	20	4	3	1	1	1	30
Sex, F/M	15/19	4/6	5/2	0/2	2/0	0/1	26/30
Individuals: Deceased/alive	28/6	8/2	7/0	2/0	2/0	1/0	48/8
Individuals: Deceased at or before 2 years of age	23	6	7	2	2	1	41
Median age at death (range)	13 months (2 days–16 years)	8 months (11 days–20 years)	14 months (4 months–20 months)	6.5 months (5 months–8 months)	11 months (10 months–12 months)	5 weeks	11.5 months
Viral infection suspected (or documented)	14 (6)	3	2	2	1	0	22
Sudden cardiac arrest	15	2	6	0	2	1	26
Cardiogenic shock/heart failure	13	6	2	2	na	na	23
Arrhythmia	9	6	1	0	0	1	17
Dilated cardiomyopathy (or ventricular dilatation)	12	1	1	2	0	0	16
Cardiac fibrosis (histology or MRI)/available	15/17	5/10	3/6	2/2	0/1	0/1	25/37
Alcohol intolerance	7	4	na	na	na	na	11
Progressive neurological signs	2	2	na	na	na	na	4

^a^Two cases from family 6, diagnosed prenatally with biallelic *PPA2* variants but without clinical features, are not included as affected individuals, while one individual with the same pathogenic biallelic variants of their deceased siblings within family 16 remains unaffected at 10 years of age.

Among the 34 individuals in this cohort, 6 remain alive and 28 are deceased. Of these 28 individuals affected by sudden cardiac death, 23 died at or before 24 months of age and five died between 14 and 16 years of age. Kaplan–Meier survival curves (Fig. 1b) generated using data from this study and from five previous reports [1–5] indicate two age groups with highest mortality: one major group represented by infants below the age of 2 years (*n *= 23 from this cohort and *n *= 41 when combined with previous reports) and in adolescence (*n* = 5 from this cohort and *n* = 7 combined with previous reports). Clinical presentations were consistent with those previously reported in the literature with either sudden cardiac arrest (15/28 individuals, including all five teenagers) or acute heart failure (13/28 individuals). In the latter group of 13 individuals, dilated cardiomyopathy or ventricular dysfunction was usually diagnosed by echocardiography, rapidly followed by multiorgan failure and/or ventricular fibrillation (VF, reported for six cases). Among the 34 cases detailed here, lactic acidosis was noted in five cases and a viral test was positive in five cases. Among the five deceased teenagers who died of sudden cardiac arrest; four had been drinking alcohol less than 6 hours prior to death.

While these clinical presentations are consistent with those previously reported in the literature, we provide several novel observations from this cohort. Individual II-1 from family 10 was diagnosed prenatally with PPA2-related mitochondrial disease. Clinical observations included severe intrauterine growth restriction (IUGR), dilated cardiomyopathy, bilateral renal pelviectasis, bilateral ventriculomegaly, agenesis of the corpus callosum, and cerebellar hypoplasia. He was delivered by caesarean section at 28+4 weeks gestation, due to worsening fetal heart rate and reversed diastolic flow. He died on day 2 of life from refractory hypoxemia, pulmonary hypertension, and progressive lactic acidosis. Postmortem examination revealed dilated cardiomyopathy.

Among the six affected individuals who are alive and aged between 4 and 40 years, individual II-1 from family 12 is noteworthy having received a heart transplant for severe and rapid-onset dilated cardiomyopathy leading to heart failure at 3 years 9 months. He remains alive and well 20 months post-transplantation. Individual II-1 from family 17 is alive aged 40 years. She was diagnosed with multisystemic mitochondrial disease with cardiomyopathy at 17 years and has had a history of progressive distal weakness and bilateral foot drop with peripheral axonal neuropathy identified by electromyography, external ophthalmoplegia, and ptosis since the age of 29 years. No ataxia was noted. Brain MRI was not informative. She reported intolerance to alcohol with severe reactions such as nausea, vomiting, or muscle pain. Individual II-4 from family 4, aged 19 years old at last follow-up, also developed progressive neurological symptoms with ataxia, spasticity, distal weakness, decreased balance and coordination, dysarthria, and dysmetria evident by 14 years of age. He has been toe walking from 1 year of age, with chronic tightness in the legs and shortened heel cords. Brain MRIs at 13 and 17 years old were considered normal with no cerebellar anomaly.

In this group of living individuals, two children (II-2 from family 5, aged 4 years old and II-2 from family 18, aged 3 years 9 months) had a history of multiple VF arrests starting at 20 months and 18 months old respectively, and were implanted with a cardioverter defibrillator. Both have cardiomyopathy with dilation of the left ventricle, with myocardial fibrosis observed in individual II-2 (family 5) following cardiac MRI. Up until publication, there have been no reports that implanted defibrillators have been triggered, neither in these individuals nor the two alive in their 40s from family 1 reported in Kennedy et al. [1] Finally, one individual (II-2 from family 16) remained asymptomatic at 10 years of age with normal electrocardiography, echocardiogram and cardiac MRIs. Her two affected brothers died of acute heart failure at 18 months and 2 years of age.

Irrespective of the main clinical presentation, myocardial fibrosis was reported in 15 cases in our cohort, either at necropsy or on cardiac MRI, whereas an inflammatory infiltrate was noted in only 6 cases and always in combination with fibrosis. Thus, fibrosis may help discriminate between *PPA2*-related cardiomyopathy and viral myocarditis when individuals present with acute heart failure.

### Identification of novel *PPA2* variants associated with cardiac failure

**Fig. 2:**
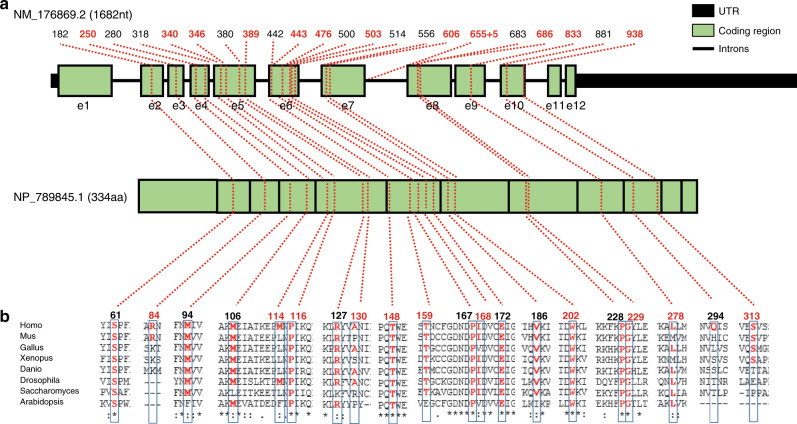
Distribution of *PPA2* variants and conservation of affected amino acids. (**a**) Genomic structure and location of all known disease-associated variants within *PPA2* (GenBank NM_176869.2) encoding the mitochondrial inorganic pyrophosphatase. Green-filled boxes represent coding exons 1 to 12. Numbers above the gene’s schematic indicate the position of complementary DNA (cDNA) variants. Red numbers indicate the novel variants reported in this manuscript. Translated protein (Genbank NP_789845.1) is represented below the gene as spliced green boxes with black borders. (**b**) Phylogenetic conservation of amino acids affected by *PPA2* missense variants is shown by multiple sequence alignment performed with the Clustal omega algorithm. Numbers reflect amino acid position. Red numbers indicate amino acids affected by novel variants reported in this paper. e exon, aa amino acids, nt nucleotides, UTR untranslated region.

Of the 12 novel *PPA2* variants (Fig. 2 and Table 2), nine are missense, two are predicted to introduce stop codons and one affects a splice site. All variants were either absent or present at a very low allele frequency in the gnomAD database. ACMG/AMP guidelines [6] were applied for variant classification of the novel (and previously reported) *PPA2* variants (Table 2). The two truncating variants: c.250C>T; p.(Arg84*) and c.938C>A; p.(Ser313*), lie in exons 3 and 10 respectively (of 12 exons in total) and are predicted to induce nonsense-mediated messenger RNA (mRNA) decay. [9] These instances of complete loss-of-function (LoF) alleles and the absence of common or homozygous LoF variants within gnomAD was taken as evidence to support LoF as a mechanism for pathogenicity. The noncoding c.655+5G>A variant lies within intron 7 and is predicted by multiple in silico prediction tools (SpliceSiteFinder-like, MaxEntScan, NNSPLICE and SpliceAI) to affect mRNA splicing but unfortunately no tissue was available to prove this in vivo; the c.655+5G>A variant is therefore classified as a variant of uncertain significance (VUS). All novel missense variants were classified as likely pathogenic.

**Table 2:** Twelve novel and ten previously reported *PPA2* variants associated with sudden unexplained death in infancy or sudden cardiac death.

Variant	Exome count	Genome count	Total freq.	dbSNP	Genomic coordinates	ACMG/AMP classification [6]	Family
**This study**							
c.250C>T;p.(Arg84*)	2 (245032)	0	0.00001	rs781655422	Chr4(GRCh38):g.105453615G>A	Pathogenic: PVS1-VSTR; PM2-MOD	15
c.340A>G;p.(Met114Val)	4 (240494)	1 (31404)	0.00002	rs375129675	Chr4(GRCh38):g.105446484T>C	Likely pathogenic: PS3-MOD; PM2-MOD; PM3-MOD	1, 2, 3^a^
c.346C>T;p.(Pro116Ser)	11 (239402)	0	0.00005	rs373735128	Chr4(GRCh38):g.105446478G>A	Likely pathogenic: PS3-MOD; PM2-MOD; PM3-MOD; PP3-SUP	8
c.389C>T;p.(Ala130Val)	0	0	0	na	Chr4(GRCh38):g.105446435G>A	Likely pathogenic: PS3-MOD; PM2-MOD; PM3-MOD; PP3-SUP	15
c.443C>G;p.(Thr148Ser)	1 (243948)	1 (31340)	0.00005	rs778534602	Chr4(GRCh38):g.105438035G>C	Likely pathogenic: PS3-MOD; PM2-MOD; PM3-MOD; PP3-SUP	12
c.476C>T;p.(Thr159Met)	12 (243948)	0	0.00005	rs752062224	Chr4(GRCh38):g.105438002G>A	Likely pathogenic: PS3-MOD; PM2-MOD; PM3-STR; PP3-SUP	7, 13, 18
c.503T>C;p.(Ile168Thr)	6 (246480)	0	0.00002	rs760824971	Chr4(GRCh38):g.105437975A>G	Likely pathogenic: PS3-STR; PM2-MOD; PM3-SUP; PP3-SUP	14^a^
c.606G>C;p.(Trp202Cys)	1 (248128)	0	0.00001	na	Chr4(GRCh38)g.105424245C>G	Likely pathogenic: PS3-MOD; PM2-MOD; PM3-MOD; PP3-SUP	11
c.655+5G>A (splice site)	1 (237254)	0	0.00001	rs1409680543	Chr4(GRCh38):g.105424191C>T	VUS: PM2-MOD; PP3-SUP	2
c.686G>T;p.(Gly229Val)	0	0	0	na	Chr4(GRCh38):g.105399134C>A	Likely pathogenic: PS3-MOD; PM2-MOD; PP3-SUP	20^a^
c.833T>C;p.(Leu278Ser)	0	0	0	na	Chr4(GRCh38):g.105396285A>G	Likely pathogenic: PS3-MOD; PM2-MOD; PM3-MOD; PP3-SUP	17
c.938C>A;p.(Ser313*)	9 (250934)	1 (31382)	0.00004	rs151331559	Chr4(GRCh38):g.105386568G>T	Pathogenic: PVS1-VSTR; PM2-MOD	10, 12
**Previous studies** [1–5]							
c.182C>T;p.(Ser61Phe)	2 (247094)	0	0.00001	rs772083375	Chr4(GRCh38):g.105456721G>A	Likely pathogenic: PS3-SUP; PM2-MOD; PP1-MOD; PP3-SUP	
c.280A>G;p.(Met94Val)	0	0	0	rs1057517679	Chr4(GRCh38):g.105449391T>C	Likely pathogenic: PS3-SUP; PM2-MOD; PM3-MOD; PP3-SUP	
c.318G>T;p.(Met106Ile)	0	0	0	rs1057517680	Chr4(GRCh38):g.105449353C>A	VUS PM2-MOD; PM3-MOD; PP3-SUP	
c.380G>T;p.(Arg127Leu)	37 (244404)	7 (31390)	0.00016	rs139076647	Chr4(GRCh38):g.105446444C>A	Pathogenic: PS3-SUP; PM2-MOD; PM3-STR; PP1-STR; PP3-SUP	6, 9^a^, 16
c.442A>T;p.(Thr148Ser)	0	0	0	na	Chr4(GRCh38):g.105438036A>T	Likely pathogenic: PS1-STR; PM2-MOD; PM3-MOD; PP3-SUP	
c.500C>T;p.(Pro167Leu)	4 (245870)	4 (245870)	0.00002	rs546693824	Chr4(GRCh38):g.105437978G>A	Likely pathogenic: PS3-SUP; PM2-MOD; PM3-SUP; PP1-STR; PP3-SUP	19^a^
c.514G>A;p.(Glu172Lys)	120 (243704)	27 (31376)	0.00053	rs146013446	Chr4(GRCh38):g.105437964C>T	Pathogenic: PS3-STR; PM2-MOD; PP1-STR; PP3-SUP	1,4,5,6,7,10,1113,16,17,18
c.556G>A;p.(Val186Met)	0	0	0	na	Chr4(GRCh38):g.105424295C>T	Likely pathogenic: PS3-SUP; PM2-MOD; PM3-MOD; PP3-SUP	
c.683C>T;p.(Pro228Leu)	49 (242474)	9 (31366)	0.00021	rs138215926	Chr4(GRCh38):g.105399137G>A	Pathogenic: PS3-SUP, PM2-MOD, PM3-MOD, PP1-STR, PP3-SUP	4, 5, 8
c.881A>C;p.(Gln294Pro)	0	0	0	rs1057517678	Chr4(GRCh38):g.105386625T>G	Likely pathogenic: PS3-SUP; PM2-MOD; PM3-MOD; PP1-MOD; BP4-SUP	

Variant nomenclature relates to coding sequence NM_176869.2 and protein sequence NP_789845.1. Exome and genome counts and frequencies calculated in gnomAD v2. Variant classifications were carried out according to ACMG/AMP guidelines. [6].

*MOD* moderate, *na* not applicable, *STR* strong, *SUP* supporting, *VSTR* very strong.

^a^Indicates the variant occurred in the homozygous state.

For eight of the nine novel missense variants, in silico predictions are categorized as probably damaging in PolyPhen-2 and deleterious or damaging in SIFT: p.(Pro116Ser), p.(Ala130Val), p.(Thr148Ser), p.(Thr159Met), p.(Ile168Thr), p.(Trp202Cys), p.(Gly229Val), and p.(Leu278Ser), with high CADD scores of 24.5, 24.2, 32, 27.3, 26.2, 32, 25.2, and 28.4 respectively, due to their high conservation (Fig. 2). Although a c.442A>T;p.(Thr148Ser) variant has been reported previously [5] as a VUS, another variant (c.443C>G) giving rise to the same amino acid substitution is found in our cohort. Functional studies presented here, relating to the impact of the p.Thr148Ser substitution upon PPA2 activity, provide further evidence of pathogenicity, with both variants being classified as likely pathogenic (Table 2).

The c.340A>G; p.(Met114Val) substitution is not strongly predicted to disrupt protein function in silico, with a CADD score of 2.8, a PolyPhen-2 prediction of benign, and a SIFT prediction of damaging. The methionine at this location is poorly conserved across mammalian and vertebrate evolution, although the hydrophobicity at this residue is conserved: most species instead have a leucine residue at this site. Only plants differ markedly, with an aromatic residue here (phenylalanine; Fig. 2). Therefore the significance of the sidechain difference between leucine and valine may be critical. We note that the region immediately surrounding Met114 is highly conserved through vertebrates. This variant was identified (or inferred present) in six individuals with early cardiac failure and death before 12 months from families 1, 2, and 3, in either the homozygous or compound heterozygous state.

Of note, the most frequent pathogenic *PPA2* variant is c.514G>A; p.(Glu172Lys), identified in 11 of the 20 families in our cohort, and has been reported in several families in previous studies. [1–3, 5] Recombinant human PPA2 containing this variant displays a 90% reduction in enzyme activity. [1] This variant has a gnomAD allele frequency of 0.00053, and has never been seen in the homozygous state either in gnomAD or in patients documented in the literature.

### Functional analyses of recombinant mutant proteins

**Fig. 3:**
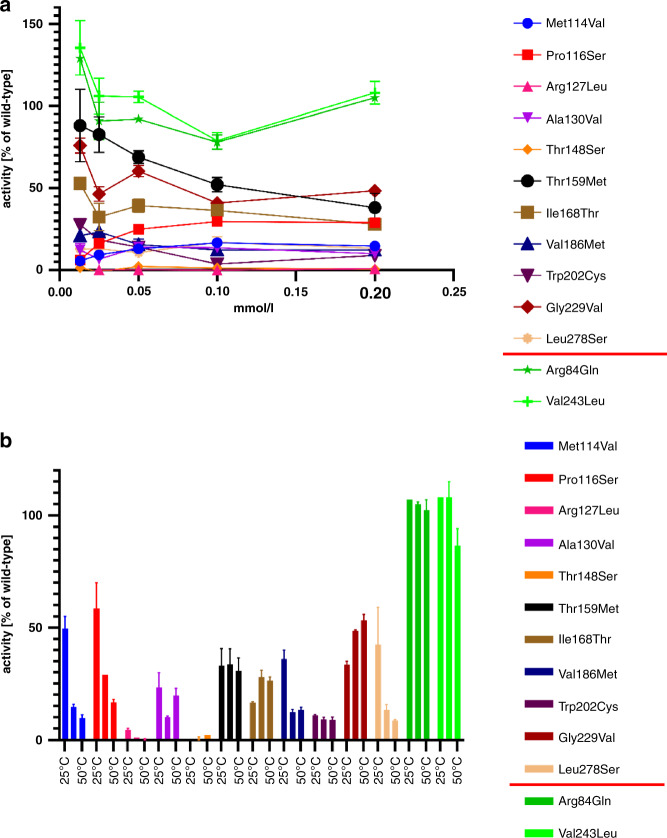
Enzyme activity of recombinant PPA2 variants. (**a**) The percentage of activity of the recombinant PPA2 relative to recombinant wild-type PPA2 at 37 °C is indicated at different pyrophosphate concentrations along the *x*-axis. At least three replicates were performed for all conditions. Two *PPA2* variants (Arg84Gln and Val243Leu), which are found in homozygosity in the gnomAD database, are shown in shades of green and serve as positive controls. Error bars show the standard error of the mean (SEM). (**b**) Enzyme activities of the recombinant proteins were determined at different temperatures (25 °C, 37 °C, 50 °C) with a pyrophosphate concentration of 0.2 mmol/l. Three replicates were carried out per recombinant enzyme, per temperature. The two *PPA2* variants in shades of green serve as positive controls. Error bars show the SEM.

To test the in silico pathogenicity predictions described above, and to explore whether correlations could be made between genotype and clinical presentations in our patient cohort, we characterized the novel *PPA2* variants identified: p.(Met114Val), p.(Pro116Ser), p.(Ala130Val), p.(Thr148Ser), p.(Thr159Met), p.(Ile168Thr), p.(Trp202Cys), p.(Gly229Val) and p.(Leu278Ser), and two from our previous work, p.(Arg127Leu) and p.(Val186Met), by measuring mitochondrial pyrophosphatase activities of purified recombinant proteins harboring these variants (Fig. 3). PPA2 enzyme activity was determined at 37 °C with varying pyrophosphate concentrations (Fig. 3a) and at a PPi concentration of 0.2 mmol/l at three temperatures (25 °C, 37 °C, 50 °C, Fig. 3b). Enzyme activities were significantly decreased in comparison to wild-type controls for all variants tested. Although relatively uncommon, we observed two homozygous *PPA2* missense variants in the gnomAD database which we presumed to be benign variants; predicted to result in the substitutions p.(Arg84Gln) and p.(Val243Leu). These were also cloned and expressed in *E. coli* as positive controls, as their presence in the database suggested they would not adversely affect PPA2 function. Both resulted in PPA2 enzyme activity similar to wild-type (Fig. 3b), illustrating the specificity of the assay and further establishing the impact of affected individuals’ substitutions tested in this study (and where relevant, previous ones).

Apart from controls, the highest residual PPA2 activity was found for the p.(Thr159Met) variant (40% to 50% of residual activity at PPi substrate concentrations of 0.10 and 0.20 mmol/l), which was identified in three individuals who survived the first 2 years of life, two of whom lived into their teenage years. All three harbored p.(Thr159Met) in a compound heterozygous state with p.(Glu172Lys), the most frequent *PPA2* variant, with a previously reported [1] low residual activity of approximately 5% to 10% compared to wild type. The variant predicted to encode the p.(Gly229Val) substitution also showed relatively high residual activity. It was identified as homozygous in individual II-1 from family 20, who presented with heart failure as a neonate with respiratory distress and bilateral vocal cord palsy. This individual died at 3 months old of cardiogenic shock. Microarray analysis showed uniparental disomy of chromosome 4.

Interestingly, the only novel variant without strong pathogenicity predictions in silico, p.(Met114Val), displayed a reduction of activity similar to several other variants (Fig. 3a), and classification using ACMG/AMP guidelines predicted the substitution as likely pathogenic (Table 2). The lowest enzymatic activities were observed for the p.(Arg127Leu), p.(Thr148Ser) and p.(Trp202Cys) mutants. The p.([Trp202Cys];[Glu172Lys]) combination is found in family 11, in which two siblings died at 43 and 45 days of life from severe infantile onset cardiomyopathy. p.(Arg127Leu) was found in combination with p.(Glu172Lys) in families 6 and 16. For family 6, an affected 12 month old child (II-2) presented with sudden death. Within family 16, two children died at 18 months and 2 years old respectively, from cardiac failure, while one sister is alive and asymptomatic at 10 years of age. In family 9, two sibs homozygous for p.(Arg127Leu) died in their teens of sudden cardiac arrest after ingestion of alcohol.

Together, genotype–phenotype correlations are difficult to establish as the same variant combinations within a family can produce variable clinical outcomes. The role of external tissue or cellular stressors such as alcohol or infection may impact the disease course of individuals carrying pathogenic *PPA2* variants. Consistent with this hypothesis, several *PPA2* variants showed a decrease of relative enzyme activities from 25 to 37 °C (Fig. 3b). This observation may correlate with symptoms appearing in patients during viral infections, a factor noted previously by the studies of Kennedy and colleagues. [1]

## DISCUSSION

In this study, we report a cohort of 34 individuals with biallelic *PPA2* variants from 20 families, enlarging the number of reported patients to 56 from 30 families worldwide. We expand the phenotypic spectrum ascribed to PPA2 dysfunction, report two truncating variants, and highlight the pathogenicity of previously undescribed missense variants using functional assays.

The most common clinical presentation remains sudden unexpected cardiac arrest, reported in all of the deceased teenagers and half of the children who died before their teens, while the other half presented with dilated cardiomyopathy, cardiogenic shock, or arrhythmia before death. Six affected individuals are alive, aged from 4 to 40 years. Clinical evaluation shows them to be variously symptomatic with both cardiomyopathy and peripheral neuropathy (1/6), symptomatic with neurological signs only (1/6), symptomatic with heart transplantation for severe cardiomyopathy (1/6), symptomatic with cardioverter inserted for recurrent VF arrests (2/6), or completely asymptomatic (1/6). Focal myocardial fibrosis upon histology and/or cardiac MRI was reported in 15 of 17 cases for which data were available. Such an observation should alert physicians to possible PPA2 dysfunction regardless of the clinical presentation and even in asymptomatic individuals. We emphasize the importance of an early diagnosis for therapeutic options and genetic counseling and note the use of prenatal diagnosis for subsequent pregnancies in family 6 following the identification of biallelic *PPA2* variants in an affected individual (II-2), leading to termination of pregnancy for two fetuses.

In addition to cardiac tissue damage, we observed progressive neurological features in surviving adolescents and adults harboring a p.([Pro228Leu];[Glu172Lys]) combination of biallelic *PPA2* variants (family 4 in this cohort, and family 1 in Kennedy et al. [1]). Following anecdotes from family members (family 1 in Kennedy et al. [1]), describing coordination and balance difficulties in all their affected children, we tested the two surviving affected siblings, at age 40 (II-2) and 35 years (II-4), and noted a discrete cerebellar ataxia in both. Brain imaging has not been performed to date. These two individuals also report a sensitivity to vinegar, causing myalgia. Individual II-4 in family 4 (this report) displayed signs of progressive spastic cerebellar ataxia with distal weakness starting from 14 years of age. The remaining case presenting neurological signs is individual II-1 in family 17, with biallelic p.(Leu278Ser) and p.(Glu172Lys) *PPA2* variants, who developed peripheral neuropathy, external ophthalmoplegia, and ptosis from 29 years of age. Interestingly, three of these four individuals also had experienced acute sensitivity to alcohol presenting as acute muscle pain (the fourth individual has not been exposed to alcohol). Finally, novel antenatal manifestations concerning brain development were observed in one individual (in family 10) with cerebellar hypoplasia, agenesis of the corpus callosum and partial agenesis of the septum pellucidum. However, given that singleton genome sequencing was performed in this individual, we cannot exclude the possibility that the cerebral malformations may be due to an undetected de novo pathogenic variant in another gene or genes. [10, 11]

Progressive neuropathy and cerebellar ataxia are frequent manifestations in mitochondrial diseases. [12, 13] Cerebellar hypoplasia and agenesis of the corpus callosum are less common, although have been reported in mitochondrial diseases [13] and other metabolic disorders. [14, 15] These observations are consistent with mitochondrial depletion or dysfunction affecting tissues requiring optimal levels of ATP availability such as muscle and nerve cells. [16–18] Neurological examination and follow-up are therefore recommended for surviving individuals harboring pathogenic biallelic *PPA2* variants.

This large cohort has allowed the identification of several variant combinations compatible with survival into childhood and beyond in some individuals from eight families: the p.([Glu172Lys];[Pro228Leu]) combination in families 4 and 5, the p.([Glu172Lys];[Leu278Ser]) combination in family 17, the p.([Thr159Met];[Glu172Lys]) combination in families 7, 13 and 18, the p.([Arg127Leu];[Glu172Lys]) combination in family 16 (although this genotype in family 6 resulted in death at the age of 14 months in individual II-2), and the p.([Arg127Leu];[Arg127Leu]) combination in family 9. Despite some surviving individuals in these families, families 4 and 16 also illustrate variability of intrafamilial expression. Indeed, in family 4, individuals II-2 and II-3 died of a cardiac arrest at 15 years and 23 months of age respectively, whereas their 19-year-old brother is alive with progressive spasticity and cerebellar syndrome. In family 16, two siblings died of cardiac failure at 24 and 18 months old whereas their affected sister remains asymptomatic with normal cardiac MRI and monitoring at 10 years of age. The observed intrafamilial phenotypic variability in the above cases makes it difficult to accurately assign genotype–phenotype correlations.

It is again worth noting the role of environmental triggers such as viral infection and alcohol consumption for these affected individuals.

Acute decompensation can be triggered by cellular stressors such as infection or alcohol. Infection is a well-known trigger in mitochondrial diseases, [19–21] while alcohol is not. In this series, an infectious trigger leading to a fatal episode was suspected in 14 individuals from 10 families (families 1, 3, 4, 6, 8, 15, 16, 18, 19, 20) and identification of a viral infection was confirmed in six of these cases, of which five had mild hyperthermia (Supplementary Table S3). In addition, previous studies [3, 4] reported four deaths at or before 12 months of age following viral infection symptoms in the days prior to death, without clearly documented hyperthermia. Infection induces the cellular response of hyperthermia, which in the case of PPA2-deficient individuals may mean a further reduction in residual PPA2 enzyme function. We propose that this may be the mechanism that affects their cardiac tissue, ultimately resulting in sudden death. We propose that viral infections in affected individuals may tip the cardiac muscle into ATP deficit, due to a build-up of PPi following the effects of temperature increase on the already deficient PPA2 enzyme of these individuals. Further study would be useful in this context.

Alcohol metabolism (and vinegar metabolism to some degree) produces acetic acid, which is then activated to acetylCoA with the formation of equimolar amounts of PPi. We note that vinegar has been reported to illicit myalgia in several affected individuals with biallelic *PPA2* variants (personal communication, family 1, Kennedy et al. [1]). The precise pathophysiological mechanism of how alcohol intake induces sudden cardiac arrest in the context of PPA2 dysfunction requires further investigation, and biochemical studies to elucidate the physiological pathways and more precise causes of mitochondrial dysfunction may be warranted in experimental settings such as cell cultures from patients or heterozygous individuals, induced pluripotent stem cells or animal models.

Considering PPA2’s function, lower enzymatic activity should lead to accumulation of PPi and depletion of ATP within the mitochondrion. The observation of acute onset lactic acidosis and cardiomyopathy in previous reports [1–4] favor this hypothesis, and focal myocardial fibrosis could reflect a subclinical reduction in ATP availability in some myocardial cells. A reduction of mitochondrial respiratory chain enzyme activities in fibroblasts has been reported in a number of families. [1–3] OXPHOS activities were only available for two cases in our cohort. In family 8, activities of complexes I and IV were markedly decreased in postmortem skeletal muscle from individual II-2; and in family 18, low borderline measurements for complex IV were found in a skeletal muscle biopsy from individual II-2. For the latter patient’s cardiac muscle sample, activities of respiratory chain complexes I, II, III, and IV were below 20% of control mean values, as was the mitochondrial marker enzyme citrate synthase, suggesting reduction of mitochondrial mass (Supplementary Table S2).

In conclusion, this study broadens the spectrum of inherited biallelic *PPA2* variants responsible for cardiac dysfunction, with 12 previously unreported pathogenic variants. The most frequent clinical presentation remains sudden unexpected cardiac arrest, but we report novel presentations including prenatal onset cardiomyopathy and progressive neurological disease in teenage years to adulthood. Although there is a tendency for the association of certain variants with varying severity of disease, definitive genotype–phenotype correlations are complicated by both inter- and intrafamilial clinical outcome heterogeneity, which may, in part, be due to the role of external stressors such as viral illness or alcohol catabolism. Genetic counseling in sibships is particularly difficult in these families. Survivors may be at risk of developing spastic cerebellar ataxia and peripheral neuropathy from their teenage years. At-risk patients may benefit from cardiac MRI monitoring, treatment of fever, exclusion of alcohol consumption, avoidance of vinegar and vinegar-containing foods, repeated neurological evaluation, and consideration for early heart transplantation or implantation of a cardioverter defibrillator.

## Supplementary information

Supplementary information

Supplementary Table S3

## Data Availability

We will supply our data and materials upon request.
