# Supplementary material for: PPA2-associated sudden cardiac death: extending the clinical and allelic spectrum in 20 new families
Source: Genet Med. 2021 Aug 16;23(12):2415–25. doi: 10.1038/s41436-021-01296-6 (PMC8629752; doi:10.1038/s41436-021-01296-6)
Supplement: Supplementary file 1 — Supplementary information [file 41436_2021_1296_MOESM1_ESM.pdf]

Supplementary material

Supplementary Case reports

Family 1 France

Two affected sibs born to heathy, non-consanguineous, white European parents. Individual II-1 died aged 6 months with multi-organ failure in the context of extracorporeal membrane oxygenation (ECMO) for sudden heart failure. She presented with fever, rhinitis and vomiting. Initial diagnosis was subacute myocarditis without arrhythmia (viral screening of tracheal samples was positive for rhinovirus and parainfluenza). Heart histology showed myocardial fibrosis. Individual II-2 died of severe dilated cardiomyopathy at age 9 months. He presented with vomiting, pallor and poor appetite three days before hospitalisation for acute heart failure requiring ECMO (five weeks) and a Berlin heart. Heart histology showed diffuse fibrosis and inflammatory cell infiltration in the myocardium. By WES, both affected individuals were found to be compound heterozygous for a paternal c.514G>A; p.(Glu172Lys) and a maternal c.340A>G; p.(Met114Val) *PPA2* variant.

Family 2 France

Two affected sibs born to heathy, non-consanguineous, white European parents. Both died before 1 year of age of acute heart failure with ventricular fibrillation. Individual II-1 died of acute myocarditis at 8 months of age. No histology is available. Individual II-2 died of acute heart failure at 2.5 months old preceded by paroxysmal supraventricular tachycardia and ventricular fibrillation. Histology showed multifocal necrosis of the left ventricle and interstitial fibrosis. Sanger sequencing of *PPA2* showed that II-2 is compound heterozygous for the paternal c.340A>G; p.(Met114Val) and the maternal c.655+5G>A variants. II-1 was not tested.

Family 3 India

Two affected sibs born to heathy, consanguineous, Indian parents. II-1 died at 12 months of age of acute heart failure. He presented with low grade fever, vomiting and lethargy. Viral myocarditis was suspected. II-2 died from cardiac arrest at ten months of age after presenting with diarrhoea, vomiting and lethargy. An autopsy was not undertaken. WES performed on individual II-2 revealed a homozygous c.340A>G; p.(Met114Val) variant in *PPA2*. Both parents are heterozygous. II-1 was not tested.

Family 4 USA

Three affected individuals among five siblings born to heathy, non-consanguineous, white European parents. II-2 died at 15 years of age of a cardiac arrest after vomiting several times, approximately six hours after the consumption of alcohol with friends (less than half a can of beer and a small amount of vodka). Toxicology suggested that her blood alcohol concentration was below the accepted lethal range for adults. II-3 died at 23 months of age of a cardiac arrest in the context of a mild fever illness after an episode of vomiting. Acute laryngotracheitis (Echovirus type 22 recovered from trachea, nasopharynx and small bowel) was established at necropsy. Cardiac histology for both individuals was consistent with cardiomyopathy, revealing likely patchy, focal fibrosis in II-3, and significant fibrosis and scarring in II-2. Individual II-4, aged 19 years at last follow-up, has progressive ataxia with spasticity (decreased balance and coordination, dysarthria, dysmetria) and some degree of atrophy of the intrinsic hand and foot muscles with distal weakness, evident by 14 years of age; electromyography indicated a possible neurogenic process. Brain MRIs performed at 13 and 17 years were considered normal. By WES, individual II-2 was found to be compound heterozygous for paternal c.683C>T; p.(Pro228Leu) and maternal c.514G>A; p.(Glu172Lys) *PPA2* variants. Site specific testing confirmed the same compound heterozygous familial variants in affected siblings II-3 and II-4, and two unaffected siblings were heterozygous for the paternal allele p.(Pro228Leu). This combination of variants has previously been reported in family 1 by Kennedy *et al.* (2016).

Family 5 UK

Two affected siblings born to heathy, white European parents. II-1 died at 19 months of age following ventricular fibrillation and cardiac arrest. Heart histology showed diffuse myocardial necrosis associated with scarring. Follow-up of II-2 with serial screening by echocardiogram and ECG was normal until 22 months of age when she had multiple ventricular fibrillation arrests and was resuscitated. Cardiac MRI suggested a possible cardiomyopathy with dilation of the left ventricle. A defibrillator was implanted. She remains well at four years of age. Both sibs are compound heterozygous for paternal c.683C>T; p.(Pro228Leu) and maternal c.514G>A; p.(Glu172Lys) *PPA2* variants. This combination of variants is also found in family 4 from this study and family 1 from Kennedy *et al.* (2016).

Family 6 UK

One affected proband born to healthy, non-consanguineous white European parents. II-2 died suddenly at age 14 months. He has a healthy older brother of 3.5 years. II-2 was well on the day before his death, but woke in the early hours of the next morning and vomited three times. He went back to sleep, but his parents found him face-down and unresponsive

in his cot the next morning. Post-mortem examination revealed a normal male child with growth parameters large for age (body weight 12kg, 91st centile). He had no dysmorphic features or congenital abnormalities and there was no significant pathology on external or internal examination, other than the following cardiac findings and evidence of a viral infection. His heart was enlarged and heavier for age (62g vs expected 45g), but appropriate for the proband's measurements. On histological examination there was myocarditis evidenced by an inflammatory infiltrate with myocyte necrosis. In addition there were small foci of fibrosis, which suggested that there may have been a previous heart problem that was either asymptomatic or that produced very mild non-specific symptoms. There was chronic inflammation in the trachea consistent with a viral infection. The lymph nodes showed expansion of the T zone also consistent with a viral infection. There was no significant pathology of any other organs. Since *PPA2* was considered a possible candidate gene for the cause of the sudden cardiac death, Sanger sequencing of the entire coding region and flanking intronic regions of *PPA2* was performed on a DNA sample from the proband, and revealed the following two pathogenic variants: c.514G>A; p.(Glu172Lys) and c.380G>T; p.(Arg127Leu). Testing for these two variants in the patient's parents revealed biallelic inheritance of the two variants. Predictive testing in the proband's healthy brother confirmed the presence of only one variant allele and prenatal diagnosis was performed in three further pregnancies in the family, which identified two fetuses each carrying both variants, leading to termination of pregnancy.

Family 7 UK

One of two daughters born to healthy, non-consanguineous, white European parents. II-2 was in good health until she suddenly collapsed and died at 14 years of age celebrating the last day of school with friends. Autopsy showed discrete fibrosis in the right ventricle and extensive mid wall fibrosis throughout the left ventricle including the interventricular septum, anterior, lateral and posterior walls. Cardiac examination of both parents and their older daughter showed normal electrocardiography, 24 hour tape, ajmaline test and echocardiography. II-2 was compound heterozygous for the paternal c.476C>T; p.(Thr159Met) and maternal c.514G>A; p.(Glu172Lys) *PPA2* variants while II-1 was homozygous for the wild-type alleles.

Family 8 Ireland

Two affected siblings born to heathy, non-consanguineous, white European parents. II-1 developed normally until he was 23 months of age, when he experienced a viral syndrome with vomiting and diarrhea followed by a rapid cardiovascular collapse 48 hours later, and did not respond to resuscitation attempts. A post-mortem examination confirmed the

presence of adenovirus. II-2 developed normally and experienced viral infections, including varicella zoster, without complications until 22 months of age when she presented with vomiting and diarrhea and was admitted overnight to the local hospital for intravenous rehydration. When discharged the following day, she was pale and lethargic with persistent diarrhea until found lifeless in bed, and emergency services were unable to resuscitate her. Autopsy found intracellular lipid deposits in her liver, heart and kidneys, and normal fatty acid oxidation in cultured skin fibroblasts. Mitochondrial respiratory chain function was assessed in post mortem skeletal muscle and activities of complexes I and IV were markedly decreased. Exome sequencing revealed that individuals II-1 and II-2 were both compound heterozygous for paternal c.683C>T; p.(Pro228Leu) and maternal c.346C>T; p.(Pro116Ser) *PPA2* variants.

Family 9 UK

Two affected siblings born to non-consanguineous, white European parents with a family history of long QT syndrome. Molecular panel screening identified a pathogenic variant (c.1697C>A; p.(Ser566Tyr)) of *KCNQ1* in individual II-2. Because of a borderline QT interval of around 460ms, II-2 was prescribed nadolol (40 mg) daily from the age of 4 years old and remained asymptomatic although compliance with medication was a concern. She failed to attend clinics for cardiac investigations including exercise tests. At age 14 years, she collapsed at home coming back from a party where she had drunk alcohol. Cardiopulmonary resuscitation was commenced and ventricular fibrillation was noted. Rapid and professional attempts of resuscitation were given including shock, adrenaline and amiodarone. Resuscitation was stopped at arrival in hospital. Post mortem cardiac histology showed massive ischemic injury throughout the ventricular myocardium including the right ventricle, interventricular septum and left ventricle. It was noted at this time that II-1, who did not carry the *KCNQ1* variant and had a normal 12 lead ECG, had died earlier aged 16 years. On the evening prior to her death, she was thought to have consumed alcohol at a party. She was found dead in bed the next day. An extended Sudden Cardiac Death gene panel identified no pathogenic variant. Post-mortem examination showed multiple areas of extensive cardiac fibrosis. Exome sequencing indicated that both sisters were homozygous for a c.380G>T; p.(Arg127Leu) *PPA2* variant. The mother is heterozygous and the father has not been tested. In order to ascertain whether unrecognised consanguinity may be involved in the genotype, SNP microarray testing of one of the sisters was carried out, which revealed a 1.9Mb region of loss of heterozygosity (LOH) at 4q24, location 104,988,768 to 106,921,594bp, which includes the *PPA2* gene. However the total autosomal LOH detected (0.21%) was below the lower limit of 1.5%, above which would indicate a degree of parental

consanguinity. SNP array analysis therefore failed to demonstrate consanguinity as a factor contributing to the homozygous nature of the *PPA2* variant in this family.

Family 10 USA

One affected male with non-consanguineous white European parents, born via caesarean section at 28 GW + 4d, due to non-reassuring fetal heart tones and reversed end-diastolic umbilical arterial blood flow in the context of severe intrauterine growth restriction, dilated cardiomyopathy, bilateral renal pelviectasis, bilateral ventriculomegaly, agenesis of the corpus callosum and cerebellar hypoplasia. Neonatal course was complicated by pulmonary hypertension, progressive lactic acidosis and edema. His family elected for transition to comfort care on day of life 2, at which time he was extubated and died. His 32-year old primigravida mother was normotensive with a negative medical history. Autopsy confirmed agenesis of the corpus callosum and cerebellar hypoplasia associated with partial agenesis of the septum pellucidum, a structurally normal heart, but dilated congestive cardiomegaly, a fluid-filled distended pericardial sac, small pleural effusions, diffuse soft tissue edema and central venous ectasia. Histology showed acute and recent biventricular ischemic injury in the inner and mid-zonal myocardium, and mid-zonal microfoci of widened interstitium, compatible with edema and/or hypocellularity and compensatory myocyte hypertrophy. There was no fibrosis, calcification or reactive inflammation. Singleton genome sequencing identified two heterozygous variants in *PPA2*, c.514G>A; p.(Glu172Lys) and c.938C>A; p.(Ser313*), and did not identify other potential genetic contributions for his brain malformations. Sanger follow-up testing with the parental DNA revealed that the nonsense variant was paternally inherited and the missense variant was inherited from the mother. A prenatal amniocentesis clinical karyotype and microarray were normal.

Family 11 France

Two sibs, term-born to healthy, non-consanguineous white European parents and presenting severe hypokinetic biventricular cardiac dysfunction of early onset requiring mechanical circulatory support (Berlin Heart®) until death at 43 and 45 days of age respectively. Cardiac muscle histology in II-1 showed fibrosis without inflammation or necrosis. Exome sequencing identified a maternal c.514G>A; p.(Glu172Lys) variant and a paternal c.606G>C; p.(Trp202Cys) variant in *PPA2* in both affected siblings.

Family 12 USA

One affected male born full-term to healthy, non-consanguineous white European parents. The patient presented sudden onset dilated cardiomyopathy at 3 years 9 months of age with rapid deterioration requiring ECMO and a Berlin Left Ventricule Assist Device (LVAD) prior to

an orthotopic heart transplant three months later. He is alive 20 months post transplantation. He was 5 years 8 months of age at last follow-up. Retrospectively, mild motor delay with independent walking at 16 months and mild bilateral ptosis were noted. He did not have speech delay nor fine motor difficulties. Histopathology on a cardiac biopsy revealed moderate myocyte hypertrophy with extensive vacuolization, moderate interstitial fibrosis and multifocal areas of myocyte dropout. Exome sequencing identified a paternal c.443C>G; p.(Thr148Ser) and a maternal c.938C>A; p.(Ser313*) variant in *PPA2*. An additional maternally inherited variant in *MYBPC3* was classified as a VUS.

Family 13 USA

A fifth son born to healthy, non-consanguineous parents of European ancestry with no personal or family history of heart disease. At age 16 years, he collapsed as he was getting out of his chair after having drunk one or two glasses of alcohol. An emergency medical team arrived 3-4 minutes later and unsuccessfully tried to resuscitate him. He had had a normal electrocardiogram (ECG) a few months prior to his death as part of a regular screening for sport. Autopsy revealed dilated cardiomyopathy with mid-myocardial scarring that involved 30% of the left ventricle circumference and dense fibrosis on histology. There was no evidence of myocarditis, ischemic injury or storage disorder. A cardiac disease gene panel identified two heterozygous variants in *PPA2*: c.514G>A; p.(Glu172Lys) and c.476C>T; p.(Thr159Met). Sanger sequencing showed that each was inherited from a different unaffected parent.

Family 14 Canada

Three out of five siblings born to consanguineous (f=1/16) parents originating from Pakistan died of SUDI between 8 and 18 months. Death of IV-1 occurred in Pakistan and no autopsy was performed. Autopsy of IV-3 and IV-4 showed extensive biventricular myocardial fibrosis, as well as interstitial inflammation, focal hemorrhage and patchy necrosis within the left ventricle. Exome sequencing in IV-3 and IV-4 identified two homozygous, possibly pathogenic variants in two genes: *Isocitrate Dehydrogenase 3A* (*IDH3A*) c.530A>G; p.(Lys177Arg), a gene with an established role in ocular disorders and *PPA2* c.503T>C; p.(Ile168Thr); a variant consistent with the presentation. Both segregated with the disease. Parents are heterozygous for both variants. The unaffected IV-2 is homozygous for the wild type alleles for *PPA2* and *IDH3A* and IV-5 is heterozygous for both variants. Both unaffected sisters have been followed for nine years with normal electrocardiogram (ECG), signal-averaged ECG, 24-hour ambulatory ECG monitoring, exercise testing and echocardiography. Isocitrate Dehydrogenase (IDH) and PPA2 assays on frozen cardiac tissue from IV-4 showed normal citrate synthase activity compared to control and an almost

null PPA2 enzyme activity, as well as close to zero activity for IDH enzyme measurements. We assume the SUDI phenotypes were entirely the result of the *PPA2* genotypes within this family.

Family 15 USA

One affected female born to healthy, non-consanguineous white European - Hispanic parents. She was admitted to hospital at 10 days of age for decreased food intake and activity following recurrent emesis over two days. At admission she was in shock with respiratory distress, hypotonia, hypothermia, hypoglycemia, lactic acidosis, hyperammonemia, leukocytosis due to *E. coli* meningitis and sepsis and was started on antibiotics and a mitochondrial cocktail of levocarnitine, riboflavin and thiamine. Viral screening was positive for influenza A. On day eight her clinical status had improved and she could be weaned to room air but lactaturia remained. Research rapid genome sequencing identified compound heterozygosity for two *PPA2* variants of uncertain significance (c.389C>T; p.(Ala130Val) and c.250C>T; p.(Arg84*). Microarray was normal. At this time, cardiology was re-evaluated and she was found to have normal troponin (0.02), CK-MB (34.6), and normal BNP (152), non-specific T wave changes and deep Q waves in inferior leads, suggesting ventricular hypertrophy on ECG, patent foramen ovale on echocardiogram and qualitatively normal left ventricular systolic function. Holter monitor showed no anomaly. She was discharged home at 1 month of age. Repeat ECG and echo at 6 months of age were normal except for a small patent foramen ovale. At 10 months of age, she presented to the emergency room with four episodes of vomiting, pallor and sleepiness without fever and was discharged home after rehydration. Three weeks later, at 11 months of age, she presented to the emergency room with retching and decreased oral intake without fever. Her last fever was one day prior (Tmax 38°C). She had lactic acidosis (8.48 mmol/L; normal range 0.5-1 mmol/L), elevated procalcitonin, leukocytosis and thrombocytosis. She was given one dose of ceftriaxone and admitted to the pediatric intensive care unit for sustained tachycardia and tachypnea. She became pulseless before transfer to the intensive care unit. Prolonged cardiopulmonary resuscitation was unsuccessful and she died in the emergency room. Autopsy revealed cardiomegaly, myocarditis, atrophy, dropout of myocytes and fibrosis.

Family 16 USA

Two out of five siblings born to healthy, non-consanguineous white European parents presented sudden cardiac death at 24 months (II-3) and 18 months (II-5). II-3 had unremarkable prenatal and neonatal histories. At 2 years of age he developed lethargy, labored breathing, diarrhea and vomiting. He was suspected of having a viral illness and was

admitted to hospital for pneumonia with hypoxia, tachycardia and hyperlactacidemia. He was intubated and died after six days. Autopsy showed myocarditis. II-5 was a dizygotic twin with no remarkable prenatal and neonatal histories who died suddenly at 18 months of age. A few days prior to his death he was admitted to hospital because of a suspected viral illness with diarrhea, vomiting and tachypnea. Cardiac evaluation showed inverted T wave on ECG, elevated troponin and a dilated left ventricle on echocardiogram. He was put on ECMO and died of heart failure on day 14. Autopsy showed myocarditis and left ventricle dilation. Genetic testing of a panel of genes for inherited cardiac disease showed two variants in *PPA2* in both affected boys: c.514G>A; p.(Glu172Lys) and c.380G>T; p.(Arg127Leu). Sanger sequencing confirmed that the variants were each inherited from a different parent. II-2 is a 10 year old asymptomatic girl, identified as compound heterozygous for the familial *PPA2* variants. Cardiac evaluation including ECG, echocardiogram and cardiac MRI were considered normal.

Family 17 Australia

One affected adult (II-1) was suspected to suffer from a mitochondrial disease due to the association of short stature, severe cardiomyopathy at 17 years of age, peripheral neuropathy, distal weakness with bilateral foot drop and axonal motor neuropathy upon electromyography, external ophthalmoplegia and ptosis since the age of 29. Cardiac function improved but cardiac MRI detected fibrosis and the insertion of an implantable cardiac defibrillator was discussed when she was 40 years of age. She reports intolerance to alcohol intake manifesting as nausea, vomiting, muscle pain and ophthalmic pain. Her mother showed cardiac fibrosis on MRI. Genome sequencing in a research laboratory identified compound heterozygosity for a paternal c.833T>C; p.(Leu278Ser) and a maternal c.514G>A; p.(Glu172Lys) *PPA2* variant.

Family 18 Australia

A 3-year-old boy born to non-consanguineous parents of mixed Aboriginal and white European descent, presented with recurrent cardiac arrests. He was a previously well child until 18 months of age when he had his first out of hospital cardiac arrest. The second event occurred at 3 years of age. Each event occurred in the context of an intercurrent febrile viral illness and on both occasions the child was noted to be in ventricular fibrillation, requiring multiple DC cardioversions and intensive resuscitation support. Each time his echocardiogram demonstrated moderate to severe biventricular dysfunction with left ventricular dilatation. Following the first event his ventricular function had gradually returned to normal. However during the second episode he developed progressive biventricular dysfunction necessitating a prolonged period of ECMO support, followed by a protracted

recovery phase which was complicated by pulmonary haemorrhage and disseminated fungal infection. Cardiac MRI demonstrated a severely dilated left ventricle with moderate to severe systolic left ventricle dysfunction and extensive left ventricle myocardial fibrosis. His brain MRI demonstrated small gliotic foci within the frontal white matter. Following successful weaning off ECMO support, he continued to have occasional arrhythmias including ventricular fibrillation and an epicardial cardioverter defibrillator was implanted. At 4 months post event he had persistent moderately impaired left ventricular systolic function. From a neurological perspective he had residual motor deficits with persistent trunk and proximal muscle weakness. Cardiac muscle mitochondrial respiratory chain enzymology levels were all low including citrate synthase. Repeat testing on skeletal muscle showed borderline-low Complex IV. Priority trio exome sequencing detected segregating heterozygous *PPA2* gene variants, c.514G>A, p.(Glu172Lys) and c.476C>T, p.(Thr159Met), maternally and paternally inherited respectively.

Family 19 Australia

Two affected siblings born to healthy, non-consanguineous Pakistani parents. II-1 was born at term after an uneventful pregnancy and was noted to be sleepy from birth. He was admitted to hospital around 4 weeks of age for hypothermia due to cardiogenic shock and multi-organ failure with hyperlactacidemia, hyperammonia and coagulopathy. Myocarditis was diagnosed and he progressively worsened despite ECMO and expired. Screening of a panel of 200 mitochondrial genes was negative. Trio exome sequencing identified homozygosity for a c.500C>T; p.(Pro167Leu) *PPA2* variant. II-2 was born at term with normal birth parameters and required CPAP for a few hours because of transient tachypnoea. Stridor was noted from birth due to laryngomalacia. He was hospitalised at 2 months of age for respiratory distress and treated by CPAP. Supraglossoplasty was postponed due to transient ECG abnormalities and raised troponin. At 3 months of age he was alert and active with normal muscle tone and milestones although he required CPAP at night and nasogastric feeding. At 6 months of age he was admitted to hospital for close monitoring during age-related vaccinations and was put on paracetamol cover. Nevertheless he presented ventricular fibrillation arrest two days later, and rapidly died several days after that, despite ECMO. Singleton exome sequencing identified him as homozygous for the familial *PPA2* variant.

Family 20 Australia

One affected male born at term to healthy, non-consanguineous white European parents. He presented with stridor and respiratory distress due to bilateral vocal cord palsy and hepatomegaly at 4 days of life. Echocardiogram revealed a ventricular septal defect with

both ventricles of normal size and normal wall thickness with mild systolic dysfunction. Biologic screening showed elevated troponin, creatinine kinase, LDH and lactates. Despite mechanical ventilation, cardiac function worsened with ventricular tachycardia followed by biventricular dilatation and dysfunction at 12 days requiring ECMO for a week. He gradually improved and was eventually discharged home at 6 weeks of age. Extensive investigations for a metabolic or infective cause were negative. A follow-up echocardiogram was considered normal at 8 weeks of age. At 12 weeks of age and after a vomiting illness for two days he presented with severe biventricular systolic dysfunction and an asystolic cardiac arrest during sternotomy for ECMO, ventricular tachycardia and ventricular fibrillation refractory to cardioversion; supportive care was eventually withdrawn. Direct heart inspection revealed generalized enlargement with extended zones of white myocardium. A SNP array-CGH showed uniparental disomy of chromosome 4 (the *PPA2* locus lies at 4q24) and exome sequencing analysis identified a homozygous c.686G>T; p.(Gly229Val) variant in *PPA2* not previously described.

Supplementary Figure S1. Steady-state comparisons of recombinant PPA2 protein concentrations

The amount of recombinant human PPA2 protein used for the enzyme activity assays was adjusted following visual inspection of bands from Ponceau S staining and Western blotting, with equal amounts of wild-type or mutated proteins used for activity measurements.

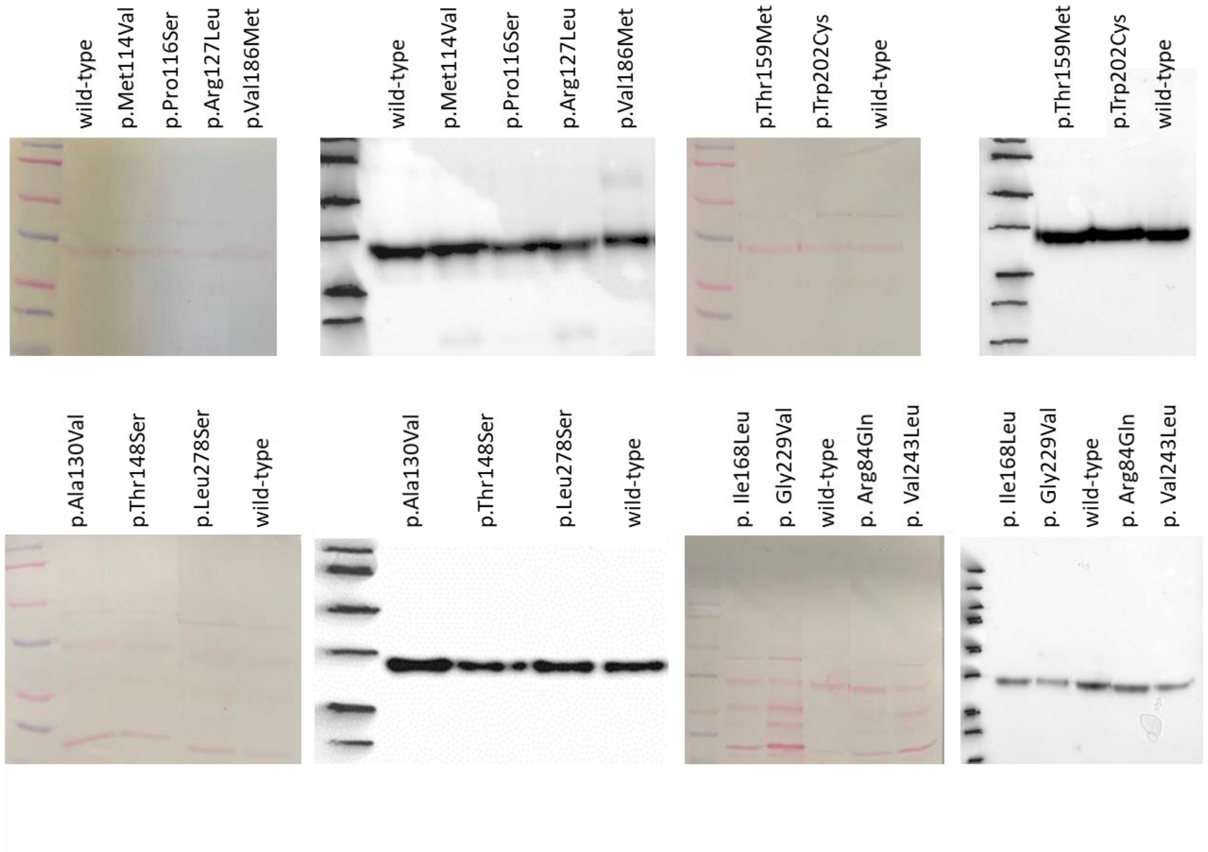

Supplementary Table S1. Mutagenesis primers

Variant	Forward primer	Reverse primer
Arg84Gln	PPA2-R84Q_F	PPA2-R84Q_R
	5'- AAGAAAGCACaAAATGATGAATATG-3'	5'- CATAGGAATGCCATTTTC-3'
Met114Val	PPA2-M114V-Q5-F	PPA2-M114V-Q5-R
	5'- CAAGGAGCCAgTGAATCCCAT-3'	5'- GTGGCAATCTCCATTTTAG-3'
Pro116Ser	PPA2-P116S-mut-F	PPA2-P116S-mut-R
	5'- TGCCACCAAGGAGCCAATGAATtCCATTAAACAAT-3'	5'- CTTTACATATTGTTTAATGGaATTCATTGGCTC-3'
Arg127Leu	PPA2-R127L-mut-F	PPA2-R127L-mut-R
	5'- TGTAAAGGATGGAAAGCTACtCTATGTGGCGAA-3'	5'- AGGGAAGATATTCGCCACATAGaGTAGCTTTCCAT-3'
Ala130Val	PPA2-A130V-Q5_F	PPA2-A130V-Q5_R
	5'- CGCTATGTGGtGAATATCTTC -3'	5'- TAGCTTTCCATCCTTTAC -3'
Thr148Ser	PPA2-T148S-Q5_F	PPA2-T148S-Q5_R
	5'- CTCCCTCAGAgTTGGGAAGATC -3'	GGTACCATAATTCCATATATAACC
Thr159Met	PPA2-T159M-mut-F	PPA2-T159M-mut-R
	5'-CCATGAAAAAGATAAGAGCAtGAACTGCTTTGG-3'	5'- CATTATCTCCAAAGCAGTTCaTGCTCTTATCTT-3'
Ile168Thr	PPA2-I168T-Q5_F	PPA2-I168T-Q5_R
	AATGATCCTAcTGATGTTTGC	ATCTCCAAAGCAGTTCGTG
Val186Met	PPA2-V186M-Q5_F	PPA2-V186M-Q5_R
	5'-AGTTATTCATaTGAAGATCCTTGG-3'	5'- TCTCCACAAGAAAGAATC-3'
Trp202Cys	PPA2-W202C-Q5_F	PPA2-W202C-Q5_R
	5'- AAACAGATTGcAAATTAATTGCTATCAATG-3'	5'- CACCTTCATCAATAAGAGC-3'
Gly229Val	PPA2-G229V_F	PPA2-G229V_R
	5'- TTCAAACCGGtTTACCTGGAAG-3'	5'- CTTCTTAACATCATCAATATCATG-3'
Val243Leu	PPA2-V243L_F	PPA2-V243L_R
	5'- ATTATATAAGtTACCAGATGGAAAAC-3'	5'- CTAAACCAATTAAGAGTAGC-3'
Leu278Ser	PPA2-L278S-Q5_F	PPA2-L278S-Q5_R
	5'- TGGAAAGCATcGCTTATGAAG -3'	5'- ACATTGATGAGTGGATTTAATAAC -3'

Supplementary Table S2. Respiratory chain enzyme activities and activity ratios in cardiac and skeletal muscle from individual II-2 in Family 18

Complexes I, II and citrate synthase (CS) are expressed as initial rates in nmol/min/mg while Complexes III and IV are expressed as rate constants in nmol/min/mg. Observed ranges are reported for n=9 skeletal muscle controls and n=7 cardiac muscle controls.

Enzyme	Cardiac Muscle		Skeletal Muscle	
	Activity	% Activity	Activity	% Activity
	(Observed Range)	(% CS Ratio)	(Observed Range)	(% CS Ratio)
Complex I	10 (64-206)	9 (56)	45 (19-72)	109 (132)
Complex II	10 (41-139)	12 (72)	57 (26-63)	126 (151)
Complex III	5.5 (15-60)	16 (92)	-	-
Complex IV	1.1 (4.2-15.5)	13 (77)	2.1 (3.3-9.1)	32 (39)
Citrate Synthase	41 (169-396)	17 (-)	107 (85-179)	83 (-)
